# Exploring resistance to implementation of welfare technology in municipal healthcare services – a longitudinal case study

**DOI:** 10.1186/s12913-016-1913-5

**Published:** 2016-11-15

**Authors:** Etty R. Nilsen, Janne Dugstad, Hilde Eide, Monika Knudsen Gullslett, Tom Eide

**Affiliations:** 1 The Science Centre Health and Technology, School of Business, University College of Southeast Norway, Postboks 235, N-3603 Kongsberg, Norway; 2 The Science Centre Health and Technology, Faculty of Health Sciences, University College of Southeast Norway, Postboks 235, N-3603 Kongsberg, Norway

**Keywords:** Ethical resistance, Welfare technology, Innovation, Co-creation, Municipal healthcare

## Abstract

**Background:**

Industrialized and welfare societies are faced with vast challenges in the field of healthcare in the years to come. New technological opportunities and implementation of welfare technology through co-creation are considered part of the solution to this challenge. Resistance to new technology and resistance to change is, however, assumed to rise from employees, care receivers and next of kin. The purpose of this article is to identify and describe forms of resistance that emerged in five municipalities during a technology implementation project as part of the care for older people.

**Methods:**

This is a longitudinal, single-embedded case study with elements of action research, following an implementation of welfare technology in the municipal healthcare services. Participants included staff from the municipalities, a network of technology developers and a group of researchers. Data from interviews, focus groups and participatory observation were analysed.

**Results:**

Resistance to co-creation and implementation was found in all groups of stakeholders, mirroring the complexity of the municipal context. Four main forms of resistance were identified: 1) organizational resistance, 2) cultural resistance, 3) technological resistance and 4) ethical resistance, each including several subforms. The resistance emerges from a variety of perceived threats, partly parallel to, partly across the four main forms of resistance, such as a) threats to stability and predictability (fear of change), b) threats to role and group identity (fear of losing power or control) and c) threats to basic healthcare values (fear of losing moral or professional integrity).

**Conclusion:**

The study refines the categorization of resistance to the implementation of welfare technology in healthcare settings. It identifies resistance categories, how resistance changes over time and suggests that resistance may play a productive role when the implementation is organized as a co-creation process. This indicates that the importance of organizational translation between professional cultures should not be underestimated, and supports research indicating that focus on co-initiation in the initial phase of implementation projects may help prevent different forms of resistance in complex co-creation processes.

**Electronic supplementary material:**

The online version of this article (doi:10.1186/s12913-016-1913-5) contains supplementary material, which is available to authorized users.

## Background

Healthcare services face vast challenges that will increase in the years to come, partly due to demographic changes including ageing populations [1, 2]. Welfare technology is viewed as one important means to meet these challenges. Implementation of digital night surveillance technologies in nursing homes and home care services has emerged as a potentially efficient way of meeting the need for monitoring persons for healthcare and safety reasons. This is an alternative to calling in on, for example, patients with dementia or intellectual disabilities, and potentially waking them up at night. However, the application and use of digital surveillance technologies in the care for vulnerable individuals generates considerable ethical debate [3–5]. Implementation of welfare technology also implies innovation and organizational change, which is often met by different kinds of resistance. Resistance can be found on individual, organizational, and institutional levels, and these levels are often inter-connected [6–8]. This paper explores if and how resistance occurs on different levels in the initial phase of digital surveillance technology implementation in municipal nursing homes and home care services.

### Implementation of innovation

Innovation has been defined as “the intentional introduction and application within a role, group, or organization, of ideas, processes, products or procedures, new to the relevant unit of adoption, designed to significantly benefit the individual, the group, or wider society” [9, 10]. This definition has become widely accepted among researchers [11, 12]. It captures many aspects of the innovation process under study, as it aims at implementing new technologies and developing new ways of working in order to benefit the individual service user and the healthcare organization. Implementation is seen as one of the four stages of innovation: dissemination, adoption, implementation and continuation [13]. The implementation stage is according to Rogers “that which occurs when an individual puts an innovation into use” ([14]:474).

Implementation of technology initiates a change process and has the potential to alter the way we work, how we organize work and the power relations in an organization. However, a large number of change initiatives fail due to unfocused and insecure management and lack of systematic project management [15, 16] or are slow to be implemented (e.g. [17–19]). The implementation phase is increasingly becoming a phase where the technology developers and the customers cooperate closely, and in the business literature it is coined as co-development of the product [20] or co-creation of value [21]. The concept of co-creation implies close and continuous interaction in the implementation phase between the innovators and developers of the technology and the customers. The technology developers may lack knowledge about the market and the users, while customers often also lack familiarity of technological language and technology proficiency. In the implementation phase of, for example, welfare technology, several knowledge spheres or epistemic cultures meet [22].

### Resistance to technology implementation

Resistance is inherent to organizational life [23, 24], and the literature on resistance stretches across several disciplines [25]. According to a recent review of research on resistance to healthcare information technologies, resistance is under-researched and multifaceted, and relatively little attention has been paid in understanding it [26]. Resistance to change has mainly been seen as an effort to maintain status quo and research has traditionally seen resistance as a negative force that must be overcome [23], and as a restraining force “that leads employees away from supporting changes proposed by managers” [27:784]. Resistance to technology implementation is ‘expected’ and can be seen as the flip side of success factors for innovation which has been emphasized in research on technology implementation in the Information Systems (IS) field (see for instance [26, 28]).

Change processes like the implementation of technology are met by several types of resistance. Resistance is found at individual, organizational and institutional levels [6–8], and these levels are inter-connected. Previous research has for instance shown that traditional organizational constellations may change as a result of technology implementation [29, 30]. Increased use of technology may change the work pattern, the division of labour and the interaction pattern. Previous research also indicates that the implementation is complicated by a lack of training and lack of interest from employees [31, 32].

Within the IS field, research on resistance concentrates on the negative paradigm, focusing on subordinates' unwillingness to implement decisions made by the management [33, 34]. Resistance occurs if threats are perceived from the interaction between the object of resistance and initial conditions [33]. Resistance creates friction, which has negative connotations and may complicate the implementation process. Friction is however also an antecedent to change [35]. As the implementation process proceeds, the users are likely to make moderations to the set of initial conditions or the subject of resistance, based on their experience with the technology. Hence the nature of the resistance will change through the implementation process [33], and resistance is not considered as purely harmful. A further example is the notion of productive resistance [23]. Productive resistance builds on the notion of resistance as a way of coproducing change and “refers to those forms of protest that develop outside of institutional channels” [23:801].

In this study, we investigate how resisters think, how they understand their own resistance and what resisters do “rather than seeing resistance as fixed opposition between irreconcilable adversaries” [23:801]. This resistance behaviour is categorized by Coetsee [36] as apathy, passive resistance, active resistance and aggressive resistance.

### Resistance to technology implementation in healthcare

Resistance to increased use of technology in healthcare is still considered to be under-researched [26, 29]. Lluch states in a review article on health information technologies (HIT) that “more information is needed regarding organizational change, incentives, liability issues, end-users’ HIT competences and skills, structure and work process issues involved in realizing the benefits from HIT” [31:849].

Furthermore, the healthcare field is not *one* field, and healthcare technology consists of a wide range of technology. Within the healthcare field, hospitals have often been the preferred empirical setting (see for example [33, 37, 38]), and physicians are the preferred actors under study (see for example [18, 37]). The municipal healthcare setting differs from that of a hospital, especially due to the organizational and structural elements of the municipality itself. The municipality is more complex and consists of several organizations, weakly tied and embedded in the larger municipal organization. Still, the levels and the various actors and units within the greater municipal organization are linked through the tasks and the users of the services. Further, the focus on patients’ interests in healthcare in general and concerning the increased use of technology, in particular, has led to focus on the groups who need to collaborate in order to implement technology [39].

Based on their studies of the implementation of information technology (IT) in hospital settings, Lapointe and Rivard [33] identified five basic components of resistance: Resistance behaviours (from passive uncooperative to aggressive), the object of resistance (the content of what is being resisted), perceived threats (negative consequences that are expected implications of the change), initial conditions (such as established distributions of power or established routines) and finally the subject of resistance (the entity, individual or group, that adopts resistance behaviours). They propose a dynamic explanation for resistance to the implementation of technology. The resistance behaviours result from the nature of perceived threats on various points in the implementation process. Depending on what triggers the resistance behaviours, new threats and consequently, new resistance behaviour emerges. The perceived threats and the resistance behaviour can be found at an individual and group level. In this article, we recognize the five basic components of resistance identified by Lapointe and Rivard, and define resistance descriptively as behaviours (attitudes, acts and omissions) that obstruct or interfere with the process of co-creation and organizational change.

### The case of Digital Night Surveillance

The innovation project at hand is called “Digital Night Surveillance”, which is a government funded project where five municipalities, both rural and urban, work with a network of technology developers to develop and implement the use of sensors and digital communication in nursing homes and home care services.

The project entailed service development and technology development in a co-creation process [21, 40] within a triple-helix inspired network [41], consisting of (1) a network of small- and medium-size technology enterprises (SMEs), (2) municipal health and care services, and (3) a university research group [42]. The overall aim was to develop and implement the best possible solution to the challenges of night surveillance, in order to enhance security and quality of care for the service users within the municipalities’ limited resources [29, 43]. The co-creation and implementation process was facilitated by a professional manager or “orchestrator” [42].

The technology to be implemented included sensors on doors and in electronic security blankets (on mattresses) used during the night. A web-based portal facilitated communication via traditional PCs as well as mobile devices, such as tablet computers and smartphones. Most of the municipal services already had some welfare technology installed, such as alarm systems. The novelty of the new system was tied to the web-based portal into which different technological applications could be connected and administered. In this way, technology in different categories and from different producers could function together and be programmed and adjusted to the individual patients’ needs. Alterations could be made based on for instance variations in needs during the day or due to the progression of a disease. An alarm went off when an incident happened. The system was programmed to send alarm messages to dedicated personnel, and they received the alarm on either a smartphone, pad or PC, or a combination of these. They ‘signed out’ the alarm as they checked on the patient.

The implementation project involved a large number of stakeholders, and the study of resistance involved exploring some of these. Data in this study comes mainly from the healthcare providers on the night shift, managers on various levels in the municipalities and healthcare institutions, and the technology developers, who also installed the equipment and trained the healthcare providers. Furthermore, the following stakeholders were involved and/or affected by the project: IT service staff, patients and families.

The home care services and the nursing homes included in the project had primary users in need of night supervision. The residents of the nursing homes suffered from dementia, and tended to get up at night and wander around, which has been described as one of the most challenging behaviours to manage [44]. Night surveillance in one form or another (face-to-face or technology based) was necessary to detect “night wanderers” and guide them back to bed in order to avoid confusion and anxiety, avoid the risk of falling and injuries, and protect other residents from being disturbed and frightened at night. In the Digital Night Surveillance project, sensors in blankets and on doors detected and sent a signal if the patient left the room. The patients did not actively use the technology; rather the users were the healthcare providers.

The participating municipalities identified a need for innovation in order to ensure safety at night for the service users. Then entered into a contract with a network orchestrator, a network of technological SMEs and a science centre for health and technology in a university, in order to run an implementation project, which included both municipal home care services and nursing homes. The initiative came from the empirical field itself.

## Methods

### Aim and study design

The aim of this study was to explore resistance to implementation of welfare technology in five municipalities in Norway. The design was explorative and draws on a longitudinal single-embedded case study [45] with elements of action research. The study was carried out during 2013 and 2014.

A case study is suitable for an explorative, in-depth study of contemporary events in its real-life context [45]. The case was a project, organized with sub-projects in each of the municipalities, with a local project manager on site. The research took a multi-stakeholder perspective as both the technology developers in the business network, who also install the technology and train the healthcare providers, and the healthcare providers, on various levels of the homecare services and nursing homes, were included in the study. The healthcare providers are the actual users of the technology and are defined as the users in our study. The study does not include data from the end-users.

Three main action research elements were applied: 1) researcher participation in the project design and planning activities, 2) researcher participation in (and by occasion also facilitation of) knowledge sharing and reflection processes during workshops and meetings, including presentation of preliminary research findings, and 3) using focus group interviews not only to collect data but also to stimulate critical reflection on the co-creation and implementation process [46, 47].

Table 1 gives an overview of the longitudinal design, the timeline, the technology, the users and the data collection methods.

**Table 1:** Design and data collection methods – an overview

Stake-holders	Technology	Research activities			
		Q3 2013	Q4 2013	Q1 2014	Q2 2014
Municipality 1	Sensor technology Alarm system Web-based portal Installations: 8	EP WS PO FG	WS PO II	WS PO	WS PO
Municipality 2	Sensor technology Alarm system Web-based portal Installations: 11	EP WS PO FG	WS PO II	WS II PO	WS PO
Municipality 3	Sensor technology Alarm system Web-based portal Installations: 9	EP WS PO FG	WS PO II	WS PO	WS PO
Municipality 4	Sensor technology Alarm system Web-based portal Installations: 4	EP	WS PO	WS PO	WS PO
Municipality 5	Sensor technology Alarm system Web-based portal Installations: 2		EP WS PO	WS PO	WS PO
Suppliers		FG WS	WS	FG WS	WS
Participants in each workshop		24	33	17	32

Abbreviations: *EP* Entered the project, *II* Individual interviews; *FG* Focus group interviews; *PO*: Participatory observation; *WS* Workshops

### Data collected

The main sources of qualitative data were semi-structured interviews, both individual and focus group interviews, and observations in workshops and meetings. Altogether, data were collected through nine individual interviews, three focus group interviews and observations on site and in four workshops. In all, about 50 individuals (including the five researchers) took part in the workshops and meetings. The researchers facilitated some of the workshops in order to stimulate co-creation and the production of process data. Twenty-one individuals were interviewed, both healthcare providers (from all five municipalities) and technology developers. All interviewed informants participated in two or more of the workshops. Some of the participants in the focus groups were also interviewed in-depth individually. All participants consented to participation in the research study.

The selection of informants from the municipalities for the individual interviews was aided by the project managers. The inclusion criteria were employees working as either project manager, middle manager or night healthcare provider. Eight women and one man were interviewed in the period from September 2013 to November 2014. Four technology developers, all male, participated in a focus group interview in January 2014. The focus group method was in line with the methodology used in the project itself, which used the workshops as an arena for orchestrated interaction, collective reflection, knowledge sharing and innovation of services [42], thereby the interviews were an arena for co-creation in themselves [48]. The in-depth interviews followed a semi-structured interview guide (Additional file 1) [49, 50] and were carried out as conversations. An interview guide was used as a checklist at the end of the interview to ensure that all planned topics were included. The first two focus group interviews with healthcare providers from three of the municipalities were performed as part of a workshop arranged early in the implementation phase, and were conducted by four of the researchers. The third focus group interview was conducted by two of the researchers with central representatives from the network of technology companies. The focus group interviews were conducted face-to-face and lasted for about 90 min each. Both the in-depth interviews and the focus group interview were digitally recorded and transcribed verbatim.

### Data analysis

Data from the interviews and observations were analysed and interpreted as inspired by Kvale’s description of the bricolage approach to data analysis [49]. Analysing data based on bricolage involves the use of various techniques and concepts during the process. We also used researcher triangulation [51], which meant that the whole research team with members from various fields such as organization and innovation studies, sociology, psychology, nursing, healthcare research and ethics, took part in the analysis and interpretation process. The main reason for choosing a researcher triangulation approach was the need for different perspectives to understand the complexity of the innovation and co-creation process, involving five different municipalities, including different professional roles, service designs, IT systems, and local decision-making procedures.

As a first step, following the description of analysis by Kvale and Brinkmann [49], the transcribed texts from the interviews were systematically read through in a naïve manner. A reflexive, open-minded and inductive reading was pursued, as well as grasping the intuitive meaning of the text as a whole and to interpret the participants’ experience and descriptions of the implementation of welfare technology. The themes in the analysis arose in an iterative process between reading and interpreting by several researchers, in order to find meaningful units and then themes according to the research question [49, 52].

Threats to validity were met by cooperating within the research team in all phases of the research project, which ensured an open discussion as well as deep knowledge of the context. The reliability of the study was strengthened through researcher triangulation and continuous contact with the network. Threats to reliability have further been met by describing the research approach in detail.

## Results

At the outset, there were few signs of resistance among the participants. As the process moved on, various forms of resistant behaviour emerged, from scepticism of the usefulness and the functionality and safety of the technology, to both passive and more active uncooperative attitudes towards the change of initial conditions, such as established routines, practices and technological infrastructure. The perceived threats were often communicated indirectly, and not always easy to identify, but in many cases, they were associated with technological instability, feelings of uncertainty and concerns for the quality of care. Resistance was found in different groups of participants and on different levels of the municipal organization. Four categories of resistance with several subcategories were identified, as laid out in Table 2.

**Table 2:** Categories of resistance

Main categories	Subcategories
Organizational resistance	• Resistance to change in established routines • Resistance to necessary competence building • Systemic resistance to communication across groups and professions • Management resistance to participatory processes
Cultural resistance	• Resistance due to language differences • Resistance due to a clash of professional cultures • Resistance against the role as co-creator
Technological resistance	• Healthcare providers’ resistance to technology • Resistance represented by IT infrastructure • IT support staff’s resistance to innovative practice
Ethical resistance	• Resistance due to patient safety issues • Resistance due to concern for the quality of care • Resistance due to patient privacy and dignity issues • Resistance due to issues of justice

In the following, the findings will be presented in more detail and exemplified, starting with organizational issues.

### Organizational resistance

#### Resistance to change in established routines

The surveillance technology was primarily introduced on the night shift, and only the night shift personnel were trained to use it. Usually, the employees worked either only night shifts or only day/evening shifts, and there was only brief contact between the shifts. The use of the technology appeared to demand a closer cooperation between the shifts. For instance, there was a need for the evening shift to prepare the technology while the patients were still awake. A night shift worker said: *“We need to have good cooperation with them, so that the mattresses are placed correctly in the evening and that they are switched on the way they are supposed to.”* Another night shift worker put it this way:

*The day shift must make sure that things work, do things well, so that I can do a good job. I cannot ask the patients to wake up and get out of bed so that I can check that everything is OK in bed. That would be stupid.*

The needs for adjusted routines and better communication and cooperation between day/evening and night shifts were soon recognized. However, both project managers and healthcare personnel experienced a lack of interest and support from the responsible middle managers and unit leaders or ward nurses. As one of the project managers answered when asked whether the unit leader had taken an active role in the project*: “No, she has barely participated and does not take the role. And she feels it is fine that I have that role”.*

This lack of managerial interest and omission to make the necessary adjustments to established routines (which was beyond the authority of the project leaders) may be interpreted as a passive form of organizational resistance to change, which interfered with, and to some degree obstructed, the process of co-creation and implementation.

#### Resistance to necessary competence building

The day shift did not receive any training in how to prepare and use the technology, and would hear about the project only through information in staff meetings. The need for training of the day shift personnel was soon recognized by the project leaders and the other participants, but the responsible unit leaders did not arrange for such. The lack of interest from the management in competence building across shifts resulted in a poor understanding of the project and the technology on the part of the day shift. One of the personnel working night shift declared:

*I feel that they do not understand any of this. It is a «night-shift-thing». (…) and I do not think they follow up, because it is never talked about. So I hoped we could have a more thorough conversation about this, not just two minutes in the staff meeting.*

#### Systemic resistance to communication across groups and professions

In addition to the lack of communication and cooperation between shifts, a more general issue emerged concerning communication, knowledge transfer and organizational learning. Communication channels across organizational levels, units and groups of professions within the complex municipal system were scarce. Those involved in the implementation of the surveillance technology lacked sufficient information about, for example, potential risks. Accordingly, this was an issue in workshops and inter-municipal meetings. However, not everybody involved could attend the workshops, and some groups – such as the cleaning staff – were not thought of as having a role in the implementation process. An example of an unforeseen risk, which proved to be a problem, was that cleaning personnel – not being sufficiently informed – on occasions moved electronic plugs and equipment in order to clean behind desks and in the corners. Breaking the electrical circuit might have the effect that sensors or communication devices shut down, and the error had to be detected before the system could be made functional again. The lack of communication channels across groups, levels and professions may represent an organizational resistance that made it difficult to prepare for unexpected errors that might obstruct or interfere with a successful implementation and use. During the workshops, it became clear that the procedures and written instructions had to include more groups than initially thought of.

#### Management resistance to participatory processes

Little by little it became clear that neither the steering group nor the responsible municipal leaders or their central IT support departments had taken sufficient measures to ensure that the necessary infrastructure was in place to serve the participating homecare units and nursing homes. It appeared that the municipalities’ IT support departments had not been included in the initial phase of the project. This was in spite of the well-known fact that the innovation technology in question required a stable technological infrastructure in order to work. If the IT support department was included, this happened at a late stage in the planning process or in the implementation process itself. Since the initiation of the implementation usually was run on the administrative level, and the crucial role of the municipal IT infrastructure would have been easy to foresee, the omission to involve the IT departments may be interpreted as a passive form of leadership resistance to collaborative and participatory processes, putting the project at considerable risk.

### Cultural resistance

The nature of the implementation project required close collaboration and interaction between different groups coming from different organizational cultures, such as the technology developers, the healthcare providers and the municipal IT staff. This collaboration was a field for learning for all parties, but also a source of resistance, that challenged established in-crowd language, professional roles, administrative routines, distribution of power and decision-making responsibilities.

#### Resistance due to language differences

There was a noticeable difference in vocabulary between the technology developers and the healthcare personnel. One healthcare provider put it this way: *“I feel they miss out on the language that they use – or what do you call it? Terminology?”*. The language gap was recognized also by the technology developers, but hard to bridge. One of them explained it as a question of awareness:

*We still have a tendency to use words and concepts from our world that we use on a daily basis, that we are actually not aware of that we use, but we can see that their eyes become glassy. And if they do not understand, they do not say so. It is a challenge.*

#### Resistance due to a clash of professional cultures

Communication problems between the technology developers and the healthcare providers went deeper than language only. Trained in different professional fields and focusing delivery of very different services (technological solutions vs care for vulnerable people), the cultural differences were considerable. This was observed during the first workshops. Both groups often used us–them language when speaking about each other, and initially there was some resistance on both sides to take the perspective of the other and actively enter into cooperation. An example is the technology developers’ reluctance to meet the healthcare providers’ needs for more written material on the technological procedures. This was clearly communicated from the outset, without being recognized. Instead, the developers adopted a passive uncooperative attitude, omitting to create the material needed. As one of the technology developers expressed: *“At the outset we hardly had any material at all. Because we perceived that this was intuitive and straightforward”.*

#### Resistance against the role as co-creator

Like the technology developers, it took a while before the healthcare providers understood their role as co-creators. The imperfections of the technology were a constant source of concern to them. For instance, alarms would go off when they should not, and vice versa. Most healthcare providers considered technological errors to be the developers’ problem, not a shared responsibility. Co-creation was perceived as foreign to them and to some degree also as a threat to their professional identity. However, some providers tried to encourage cooperation and to bridge the gap between them and the developers:

*It is a pilot project, and as I said to NN [technology developer], everyone has not understood that. That we should not have a negative attitude towards everything that we are testing out. We can be negative when the project is over, if nothing works*.

This clash of professional cultures was to some degree anticipated by the orchestrator, designing the workshops partly with the aim of two-way cultural translation and learning. It was a steep learning curve for both parties. The technology developers learned a lot about healthcare and started using some of the healthcare vocabulary. Likewise, the healthcare providers became more familiar with the technology and the developers’ way of thinking: “*When I am with them now I understand more what they mean and what they are talking about, because I am more into the system…*”

The communication and mutual understanding improved in the course of the project. New material was developed, the vocabulary changed, more procedures were included, and material was also customized to each municipality and to different groups of users (healthcare personnel, patients and relatives). However, this was primarily done by the local municipal project managers. They had expected the technologists to take more responsibility for improving and customizing the material. From their point of view, elements of passive resistance behaviours among the developers did not diminish.

### Technological resistance

Under the heading “Technological resistance”, we group both the resistance to the technology and the resistance represented by the technology itself.

#### Healthcare providers’ resistance to technology

To some of the healthcare providers the technology was in itself threatening. It challenged their sense of predictability, professionality and competence, which influenced their motivation to use the technology negatively. A main source of resistance was fear of not coping with the new technology. To some this was due to lack of familiarity with sensor technology and/or digital communication devices, and to others due to negative experiences with technology in the past. An example of the latter was a healthcare provider who for weeks had dreaded participation in a training session and even considered asking for a sick leave. She remembered her negative experience with the implementation of electronic patient records some years prior, when she ended up with a frozen shoulder. As the healthcare providers’ experience with the technology and the understanding of its prospects increased, however, the resistance decreased and the attitude became increasingly positive and enthusiastic. One of them expressed it this way: *“On our team, we have a positive attitude towards this. I believe many of them find this exciting.”*

#### Resistance represented by the IT infrastructure

Perhaps the most resistant subject of resistance, interfering with and to some degree obstructing a successful co-creation and implementation process, was the municipal IT infrastructure itself. In several of the municipalities, the technological infrastructure was in its infancy, and in some institutions, internet was not installed. If it was installed, it was often very unstable. As one of the healthcare providers said:

*And the fact that our network is down a lot, and the system in the whole municipality is very difficult to handle, as NN* [technology developer] *and they have said, it is very hard to handle. And that has made it very difficult for the technology developers and us. Well, it did not matter that much for us, but as the project was going to be terminated soon they needed to have it running, and it was very difficult. I did feel a bit sorry for them.*

The technology developers described it like this:

*We knew that there were differences, but when you really get out there you see how it works and a lot of things fall in place. And there are large differences in the infrastructure, some places they do not have a network at all, and do not use it for anything, no technology. Other places they use a lot.*

According to both the healthcare providers and the technology developers, the technological platform and the infrastructure did not provide the necessary stability for digital surveillance at night.

#### IT support staff’s resistance to innovative practice

The co-creation and implementation of technology in the making also required close cooperation with the central IT department and the support staff in the municipalities. The developers could experience resistance from the support staff in the form of reluctance or sometimes uncooperative attitudes, making implementation difficult. The developers themselves explained this by pointing to a contradiction in logics between the IT support whose focus was an efficient system maintenance, safety and predictability, and an innovative practice, implying co-creation and implementation of new technology:

*From a technological point of view, it is very difficult to innovate in a sector that… where there is a contradiction between running efficiently and innovation. Because… IT in the municipalities have stability as their main goal, and innovation leads to instability, at least when you want to try out brand new technology.*

One example of resistance to innovative practice was the reluctance to change established IT system routines. In most of the municipalities, there were routines for running the system updates during the night. This is incompatible with the use of digital night surveillance within the same system, because it represents a threat to the security of the patients when the system is shut down in order to run updates. The healthcare providers became aware of this routine only after they started using the new technology. A healthcare provider explained how this routine interfered with successful digital night surveillance:

*They run updates once a week, and at that time we cannot register and write reports. And when I entered to turn off the alarms, the system was down. So I could not get them turned off, so they just continued to go off. And all that was hopeless. And then my whole tool* [technology] *is wasted. And time and again they ran the updates during the night.*

### Ethical resistance

From the very beginning, healthcare providers, even individuals with a generally positive attitude towards technology and innovation, expressed moral concerns. One such concern was whether the motivation behind the project was morally good or not, if it was initiated in order to enhance the quality of care or to lower the cost*. “I find it [welfare technology] the right way to go. But the ethical part of it, that I'm concerned with. Not to do it to save money. That would be quite wrong.”* The implicitly perceived threat seemed to be an imagined future where implementation of welfare technology is a means of budget control at the expense of competent healthcare.

#### Resistance due to patient safety issues

Resistance among healthcare providers emerged also from a concern for patient safety and from fearing that the implementation of an unstable surveillance technology might cause adverse events and harm to patients. As the stability of the technology increased, however, this attitude of scepticism and resistance changed during the project period. A member of the staff put it this way:

*Thus, it [the technology] really makes the night shift feel safe. You can just watch the smartphones and see that the patient is sleeping, and we have had on-call staff at night who were very impressed.*

#### Resistance due to concern for the quality of care

Concern for the quality of care was evident from the start. Some perceived the surveillance technology as a threat to preconditions for maintaining a high professional standard, like face-to-face communication, attentive observation, tacit knowledge and professional judgement. When, for example, the healthcare providers no longer needed to enter the patient’s room at night unless the alarm on her smartphone went off, she felt like she was missing important information that she would have got if she had been physically present in the room. This included smelling and seeing the whole picture and, at times, communicating with the patient. As one informant expressed it:

*but there is something about, as I am saying, when I enter a patient room then there is something about what I see and smell and find out how things are as a whole, plus he* [the patient] *might say that today I would like to watch TV a bit longer… for example.*

#### Resistance due to patient privacy and dignity issues

There was also a concern for patient privacy and dignity and how this would be ensured. Was not surveillance an invasion of patient privacy, and a threat to privacy at work? These questions were subject to moral deliberations from the start:

*I have no problem displaying what I do at work. I rather think of the user, of … Where did the privacy go? I enter and leave the room and do my job, and am supposed to be professional. But the users shall feel that they have a private life when they enter their flat, that they are not going to be under surveillance, 'cause that is unnatural.*

In the beginning, some of the healthcare providers held the view that digital night surveillance was a threat to patient privacy and dignity. This view seemed to change, however, and the resistance that emerged to this perceived threat seemed to convert into a moral argument in favour of digital night surveillance. As the experience with the technology grew, a critical view on previous practice emerged. The argument was that ordinary, regular night visits, including observation while the patient was asleep, might represent a far more serious invasion of privacy and violation of dignity than a digital signal on the nurses' phone when assistance was needed. Digital night surveillance made it possible not to disturb the person in question unnecessarily, for instance, avoid waking him or her up at night in order to perform intimate actions, like adult diaper checks.

#### Resistance due to issues of justice

A final moral issue that was raised among healthcare providers that gave rise to some resistance to the project was the question of equal access to and just distribution of the technology. In this project the technology was not implemented on a large scale and accessible to all. Not all patients that could have benefitted from the technology had access to it, and some patients moved into nursing facilities where the technology was installed, without using it. This was sometimes hard to explain to relatives, but did not interfere with the innovation and implementation process.

In general, there was a change during the project period from scepticism and resistance, to a broader acceptance, and to some degree even enthusiasm, on moral grounds among healthcare personnel. One of the technology developers also made this observation:

*It has quite clearly been a change here, and the best example is that some years ago we were fighting against the perception that it was unethical to use technology here, that this was all about the warm hands (…) whereas now the norm is that it is unethical to not use the technology.*

## Discussion

### Four main forms of resistance – and perceived threats

This exploration of resistance to an implementation of welfare technology in municipal healthcare services has displayed a series of resistance behaviours, mostly passive and uncooperative, among different groups of agents – management, IT management, support staff, technology developers and healthcare providers. Four main categories of resistance were identified: 1) *organizational resistance*, including management resistance to participatory processes and necessary competence building, 2) *cultural resistance*, including resistance to cooperation and co-creation across professional groups, 3) *technological resistance*, including resistance represented by the municipal IT infrastructure itself, and 4) *ethical resistance*, including healthcare providers’ resistance to implementing the new technology. The resistance seemed to emerge from a variety of perceived threats, partly parallel to and partly across the four categories of resistance: a) threats to stability and predictability (fear of change), b) threats to role and group identity (fear of losing power or control), and c) threats to basic healthcare values (fear of losing moral or professional integrity).

### Implementation ambivalence

Summing up these findings, it might seem that there was a massive resistance to technology implementation. This was not the case. Except for the quite strong and persistent resistance represented by the IT infrastructure, most of the identified forms of resistance were passive more often than active, weak rather than strong, subtle rather than outspoken. Some of the initial scepticism and resistance even became the opposite, such as resistance due to moral concerns, which to some degree transformed into moral motivation and arguments for applying the new technology when the concerns were met and the technology worked safely. In addition, parallel to the variety of resistance, there were also considerable positive interest, energy and enthusiasm among the participants. In other words, the exploration of resistance to co-creation and/or implementation also unveiled that the variety of forms of resistance most often were intertwined with the opposite, a motivation to co-create and implement the technology. To various degrees throughout the project period, such implementation ambivalence characterized most of the participants, both developers, IT personnel, healthcare providers, projects leaders and municipal managers.

### Productive resistance

It seems like both resistance and ambivalence were productive as sources of creativity and co-creation. For example, the resistance that emerged from the threat of technological instability, unpredictability and lack of safety also triggered healthcare providers’ and developers’ creativity and cooperation to improve the technology and service. The healthcare providers helped co-create the technology through resisting the use of a technology that was not fully developed. Likewise, the technology developers helped co-create new service routines through resisting the acceptance of a non-technological practice. This may be characterized as ‘productive resistance’ [23]. In this project, productive resistance emerged from two elements: a technology or practice that failed and a co-creation process design that aimed to develop unfinished products or services [23]. The resistance became a constructive force that pushed the innovation process forward. The main reasons why much of the identified resistance in this project seemed to turn productive were probably 1) the use of an orchestrator, external to both of the participating ‘camps’, and 2) a workshop design, functioning as a learning network where all parties could meet regularly, share experiences and reflect openly together [53, 54]. Orchestrating the workshops as processes of ‘translation’ between the different professional cultures [55] was key to developing trust, enhancing knowledge of each other’s perspectives and making resistance turn productive.

### Organizational resistance

The classical theoretical approach to resistance in organizations has a negative outlook on resistance, seeing resistance mainly as a counter-force to power and control mechanisms [24, 27]. The active resistance acted by the municipal IT support department as well as a more passive resistance from the management in the healthcare institutions may have been motivated by the fear of losing power. This was intertwined with the “struggle” between stability, safety and predictability on one hand, and co-creation on the other. Participation in a pilot project evoked a certain resistance in itself, since the technology was under development and in need of improvement. This was the exact purpose of the project, but included nonetheless an element of dynamism and insecurity that was contrary to the services’ need for control and stability. The IT support departments, in particular, appeared to have a low degree of tolerance towards insecurity and loss of control.

### Cultural resistance

Cultural resistance refers to both the communication problems between healthcare providers and technology developers, as well as the resistance that emerged from the implementation of the project’s feature as a co-creation project [21]. Even though the innovators contributed to “promulgation and spread of novelties” [29:1], the communication difficulties appeared to be based in both the lack of shared vocabulary and in a mutual prejudice of the other sphere (technological vs healthcare). These cultural tensions as well as a mutual foreignness to co-creation [20], evoked resistance to the role of co-creator in both ‘camps’. Cultural differences and lack of redundant knowledge are challenging barriers to overcome in the implementation of technology [56], and the orchestrator who designed a translation process in both directions proved to be justified [42, 55].

### Technological resistance

Concerning technological resistance, there were two surprising findings. The first was that the municipal IT infrastructure in itself represented a serious resistance to the implementation process. From our material, the IT infrastructure emerged as perhaps the most uncooperative entity of all, a subject of resistance in its own right. This might seem strange, considering that subjects of resistance normally are individuals, groups of persons or organizations. However, the observation that an artefact can serve as a social-relational function is not new. The Actor Network Theory provides a corrective to the usual social scientific focus upon human beings by “directing attention to the significance of nonhumans in social life” ([57]:109) – in this case the IT infrastructure, obstructing the process of co-creation and innovation.

The second surprising finding was the passive resistance represented by the fact that nothing was done on the management level of the municipalities to include the IT departments at an initial stage, in order to prepare the IT system and support staff for the co-creation and implementation process. This is even more surprising considering the well-known fact that the municipal IT infrastructure would play a crucial role, and that implementation of welfare technologies is high on the political agenda. We have interpreted this omission as passive management resistance to participative processes. This finding is in line with research on collaborative innovation projects in the public sector, identifying co-initiation as a success factor, suggesting that public leaders and managers may be reluctant to co-initiation because of fear of losing power [58]. We can only speculate as to what, in this case, the perceived threat might have been – fear of losing power, financial consequences or something else. Whatever the reason might be, the finding suggests that more attention should be drawn to the importance of *co-initiation* and participative processes at an initial stage when planning complex municipal innovation and implementation projects.

The resistance from the IT support staff can be characterized as active resistance and was at times perceived by other stakeholders (healthcare providers and technologists) as aggressive [36]. For the managers, it appeared to be due to a poor understanding of their role in the implementation process [59]. The management did not take an active interest in the implementation, and their lack of interest can be categorized as a passive resistance that manifested in practice [33, 36].

### Ethical resistance

Ethical resistance refers to resistance emerging from reflection on perceived threats to basic healthcare values and professional ethics [60, 61]. Four main perceived threats were identified: 1) threats to patient safety, 2) threats to the quality of care, 3) threats to patient privacy and dignity, and 4) threats to equal access and just distribution. These findings are consistent with previous research with regard to the development and use of welfare technologies [3, 5, 62, 63]. Indirectly these moral concerns seem to represent arguments that may be found in healthcare (organizational and clinical) ethics. These are based on ethical theories, like the moral obligations to secure patients’ safety and rights (duty ethics), to consider moral implications, such as possible harm to patients' privacy, dignity, autonomy and integrity (consequentialism), and to protect one’s integrity as a morally mindful, caring and professional healthcare worker (virtue ethics) [64, 65].

Ethical resistance concerns the core of the healthcare providers’ professional practice, including how she uses her knowledge, skills and senses when she sees, touches, smells and speaks to the patient. Changing circumstances in the form of increased use of technology is perceived to alter and discipline the professional work [66], and professionals face new threats that have to be managed. These can be fear of not being a good healthcare provider or a caring institution and a threat to their identity as healthcare providers. Due to the changing circumstances, the content of the professionalism is contested.

The concept of ethical resistance might help leaders to recognize that this kind of resistance represents cues to moral concerns that have to be identified and solved in order to prevent adverse events and to help transform staff resistance into motivation. The concept might also help leaders avoid the psychologization fallacy, to confuse the ethical resistance of putting values at risk with the psychological resistance of change as a negative force that has to be overcome. It might also help leaders develop their ethical leadership skills [67], by using ethical resistance as a golden opportunity of detecting and managing moral risk and improving the moral quality of both the implementation process and final result [67].

In concluding the discussion, according to the informants, the initial resistance and scepticism of the new technology was replaced to a certain degree by a positive attitude towards implementation of the technology. We see three partly overlapping explanations for this. One might be adaptation, meaning that the healthcare providers got used to the technology and learned that it was helpful, not harmful [33, 68]. Another explanation might be ethical reflection upon the experience that the surveillance technology proved to enhance patient safety and reduce intrusions of privacy at night. A third explanation might be the facilitated interaction and knowledge sharing, including ethical reflection, during workshops and other meetings. This might have contributed both to adaptation, solutions to moral problems and a feeling of connectedness, competence and coping, factors associated with motivation [69, 70].

### Implications for practice

In planning the implementation of welfare technology in municipal organizations one should consider a) the IT infrastructure, b) co-initiation, c) translation spaces and d) use of an external orchestrator.

Managers should consider ethical resistance as productive, and promote co-creation between care personnel and technologists in order to meet the moral concerns.

### Issues for further research

In studies as the one at hand, many factors influence the context. In order to reduce complexity, we have omitted several factors. Central and important stakeholders like the patients and next of kin have not been included in the study. This is because we wanted to focus on the employees, but at the same time, we recognize the patient and his/her family as the real end user of the welfare technology. Focus on the patient and families will need to be included in future studies.

## Conclusion

This study identifies forms of resistance that appear to slow down the implementation of technology in a healthcare setting, especially resistance to participate in collaborative processes, resistance connected to the IT infrastructure and resistance arising from ethical concerns. It contributes to the body of literature on resistance to technology in a municipal healthcare setting, since the majority of extant research on resistance in healthcare has been performed in hospitals. Furthermore, the technology in question is sensor technology in combination with a web-based portal, which is also atypical for studies within the field.

Contrary to what might be expected from previous findings (e.g. [8]), we found that resistance to surveillance technology on a general note was not significant, and the healthcare providers perceived the new technology as a threat only to a low extent. In the long term, this could be explained by involvement in the co-creation process and motivated by a perception that a positive attitude towards this technology is appropriate and “modern”, rather than seeing technology in itself as a threat. The healthcare providers also appear to conceive the advantages and the future use of welfare technology.

Theoretically, the study contributes by identifying resistance categories, coining the concept of ethical resistance and focusing on productive resistance. Resistance appears to play a productive role when the implementation is organized as a co-creation process. The study has shown that resistance changes character over time and that it is not solely a negative phenomenon, as it contributes to development and innovation through the friction it creates.

## Additional file

Additional file 1: Interview guide for semi structured focus and individual interviews. (DOCX 14 kb)

## Abbreviations

EP: Entered the project
FG: Focus group interviews
HIT: Health information technologies
II: Individual interviews
IS: Information systems
IT: Information technology
PO: Participatory observation
SME: Small and medium-sized enterprises
WS: Workshops
