# Supplementary material for: Exploring resistance to implementation of welfare technology in municipal healthcare services – a longitudinal case study
Source: BMC Health Serv Res. 2016 Nov 15;16:657. doi: 10.1186/s12913-016-1913-5 (PMC5111186; doi:10.1186/s12913-016-1913-5)
Supplement: Additional file 1: — Interview guide for semi structured focus and individual interviews. (DOCX 14 kb) [file 12913_2016_1913_MOESM1_ESM.docx]

Project: Digital night surveillance, 2013 - 2014

**INTERVIEW GUIDE**

**Interview starts with “grand tour” question (around the table if focus group)**: How have you perceived your participation in the Digital Night Surveillance project?

**THEME 1: COMPETENCE**

Aim: To identify the emerging need for competence in the process of implementation of welfare technology.

1. How is the training organized? Description from A to Z: information and summons, who organized the training? What happened when you started to work with the technology? The role of the management? In your own words, what questions are asked and so on? Could you provide an example?
2. How can we characterize the communication between the technologists and the health care workers in the process of implementation? Can you describe a situation where you misunderstood each other? How do you experience this communication?
3. What barriers could be identified during training and implementation?
   1. what has been simple?
   2. what has been complicated?
   3. could you describe a situation that has been difficult?
4. How may the barriers be categorized?
5. What categories of knowledge are needed for implementation of welfare technology? Good information about the nature of the project? Practical training? What kind of knowledge has there been a lack of?

**THEME 2: ORGANIZATIONAL CHANGES**

Aim: Describe organizational changes that emerge as a result of implementation of welfare technology.

1. How would you describe the encounter with the organization and the employees (question for developers/technologists) OR with the technologists (question for health care workers)?
   1. Who have been interested in the project?
   2. Who have appeared best informed?
   3. Whom did you have the most contact with?
   4. How was the relation between the day and the night shift?
   5. The communication – what is your impression of the communication? Has it been of importance?
2. In what way does the division and organization of labor change as a result of the implementation of the new technology?
   1. Who have appeared best informed about the project?
   2. Who do you ask?
   3. What is the relation between the day and the night shift?
   4. The communication: has something changed as a result of the implementation project?
3. In what way have the tasks of the manager changed?
4. how has the project been handled by the management?
5. is the manager interested in the project?
6. is the (top) management interested in the project?
